# Occurrence and Risk Assessment of Mycotoxins through Polish Beer Consumption

**DOI:** 10.3390/toxins11050254

**Published:** 2019-05-07

**Authors:** Jan Grajewski, Robert Kosicki, Magdalena Twarużek, Anna Błajet-Kosicka

**Affiliations:** Department of Physiology and Toxicology, Institute of Experimental Biology, Faculty of Natural Sciences, Kazimierz Wielki University, Chodkiewicza 30, PL85064 Bydgoszcz, Poland; jangra@ukw.edu.pl (J.G.); robkos@ukw.edu.pl (R.K.); twarmag@ukw.edu.pl (M.T.)

**Keywords:** beer, mycotoxins, ochratoxin A, deoxynivalenol, HT-2 toxin, occurrence, risk assessment

## Abstract

Poland is one of Europe’s leading producers and exporters of beer. The study, herein, describes the measurement of ochratoxin A, deoxynivalenol, nivalenol, T-2 toxin, HT-2 toxin, diacetoxyscirpenol, and zearalenone levels in 69 Polish beers. Analytical methodologies based on high performance liquid chromatography (HPLC) with tandem mass spectrometry (MS/MS) and fluorescence detection were developed, validated, and used to perform the above determinations. The most prevalent mycotoxins were deoxynivalenol (96%), ochratoxin A (93%), and HT-2 toxin (74%), respectively. Three quarters of the samples contained at least three analytes. The mean ochratoxin A concentration was 0.057 (SD 0.065) ng/mL, and in four beer samples its level exceeded 0.2 ng/mL, a value postulated in the literature to be the maximum limit. Deoxynivalenol was found at a maximum level of 56.2 ng/mL, and its mean concentration was 17.1 (SD 9.0) ng/mL. An evaluation of the estimated daily intake (EDI) of mycotoxins from beer in different European populations was made using food-consumption data prepared by WHO. Based on the mean ochratoxin A concentration in beers, the EDI represented 0.8–1.1% of the tolerable daily intake (TDI), while in a worst-case scenario (maximum concentration) it reached 5.0–7.5% of TDI. For deoxynivalenol, the EDI was in the range of 4.1–6.0% of TDI, whereas, based on maximum values, it reached the level of 14–21% of TDI. There were no significant differences between “scenarios” in the HT-2 case (mean—5.0–7.5% of TDI; maximum—6.5–9.7% of TDI) due to the fact that its concentration was near the limit of quantification (LOQ) value taken for calculation. The significance of these results are discussed, herein.

## 1. Introduction

Beer is one of the most popular alcoholic beverages in the world, and it is consumed in large amounts in almost every country. It originates from prehistoric ages. Beer-like beverages were known in China, as long as 70 centuries before the current era. One of the beer precursor could be “braga” or “bosa”, relatively low alcoholic drinks, which were made over a large area of Europe, stretching from Poland to the Balkans [1]. Whatever its origins, the popularity of beer continues; it is one of the most consumed beverage in the world. Hence beer plays an important role in the human diet, and any foodstuff consumed in such large quantities is a potential path for ingestion of harmful substances. 

Mycotoxins are a group of around 500 toxic secondary metabolites produced mainly by the fungi of the *Claviceps*, *Alternaria*, *Fusarium*, *Penicillium*, and *Aspergillus* genera. These fungi grow on a variety of foodstuffs of both animal and plant origin. Maximum permitted levels of mycotoxins in food products are regulated by the EU [2,3,4,5,6,7] and other regional agencies. However, beer is one of a number of foodstuffs where maximum permitted levels of mycotoxins have yet to be firmly established. Poland is currently the third-ranking beer producer in Europe (Table 1) and so was the focus of this study. In 2016, Germany had the highest consumption of beer in Europe with 85.5 million hectoliters of beer consumed (104 L per person), followed by the United Kingdom with 43.7 million hectoliters (67 L per person), Spain with 38.6 million hectoliters (46 L per person), Poland with 37.9 million hectoliters (98 L per person), and France with 21.3 million hectoliters (33 L per person). The Czech Republic had the highest per capita consumption of beer in Europe, with 143 L of beer consumed, followed by Germany and Austria, with 103 L per person [8].

The possibility of mycotoxins finding their way into beer was always considered likely; species of *Fusarium*, as well as *Aspergillus ochraceous* and *Penicillium verrucosum* grow in poorly stored grain, and also contaminate growing cereal plants [9]. The latter two fungi produce ochratoxin A (OTA), which is nephrotoxic, teratogenic, immunotoxic, and a possible neurotoxin. OTA is also suspected to be the cause of chronic diseases including, Balkan Endemic Nephropathy (BEN); Chronic Interstitial Nephropathy (in North Africa); and urinary tract tumors in humans. OTA was classified by The International Agency for Research on Cancer as a possible human carcinogen (group 2B) [10]. The possibility of OTA getting into beer from contaminated grains used in brewing has been pointed out in literature. On the other hand, it is considered that OTA can persist the fermentation processes, but normally it can be reduced (up to 89%) during the malting process used in beer production [11]. Other mycotoxins likely to be found in beer are the trichothecenes (mainly deoxynivalenol (DON)), and zearalenone (ZEN) produced by the *Fusarium* species. Levels of DON might decrease or increase, depending on the process stage and the parameters of beer production [12,13]. The main source of mycotoxins in beer seems to derive from contaminated barley and malt feedstocks. The presence of OTA and *Fusarium* toxins in raw materials intended for malting and brewing, has previously been examined worldwide [14,15,16]. Similarly, beer contamination with mycotoxins has been the subject of numerous studies. OTA levels in beer might vary from pg/mL [17,18] to µg/mL in traditional African beers [19]. *Fusarium* derived mycotoxins do not usually exceed a value of several dozen ng/mL [12,20,21,22]. Higher values for mycotoxins have been recorded for craft beer samples, where processing is less controlled [23]. 

The objective of this study was to determine ochratoxin A, as well as trichothecenes group B (deoxynivalenol and nivalenol) and group A (T-2 toxin, HT-2 toxin, and diacetoxyscirpenol), and zearalenone, in beer produced by the main Polish breweries, and to evaluate the exposure of European populations to those compounds, considering the high position of Poland in the beer export, and taking into account the European consumption data. For this purpose, two analytical methods based on HPLC were validated.

## 2. Results and Discussion

### 2.1. Method Validation

Six-point calibration curves were constructed, based on standard solutions, with the concentation ranges of: DON: 12.5–503 ng/mL; NIV: 13.1–525 ng/mL; T2: 4.7–188 ng/mL; HT-2: 4.7–188 ng/mL; DAS: 3.6–145 ng/mL; and ZEN: 1.3–55.6 ng/mL, containing fixed amounts of internal standards ((ISs); except for the OTA) analysis. The correlation coefficients (*r*) of all analytes determined by the sum of least squares regression analyses, were higher than 0.995. To evaluate the experimental accuracy and precision, recovery experiments were carried out, using a blank lager beer (a sample with the concentration of the mycotoxins of interest lower than the detection limits) as a representative sample matrix. The recovery was calculated using the blank beer samples spiked with seven mycotoxins, at three different levels, with triplicate analyses conducted for each level. The limit of detection (LOD) and the limit of quantification (LOQ) were fixed at the mean concentration, at which the signal to noise ratio (S/N) equaled 3 and 10, respectively. For quality control, both a blank sample and positive samples (*n* = 3, ~every 20 samples) were applied in control spiking experiments, giving the recovery rate >70%. Validation data are summarized in Table 2.

**Figure 1:**
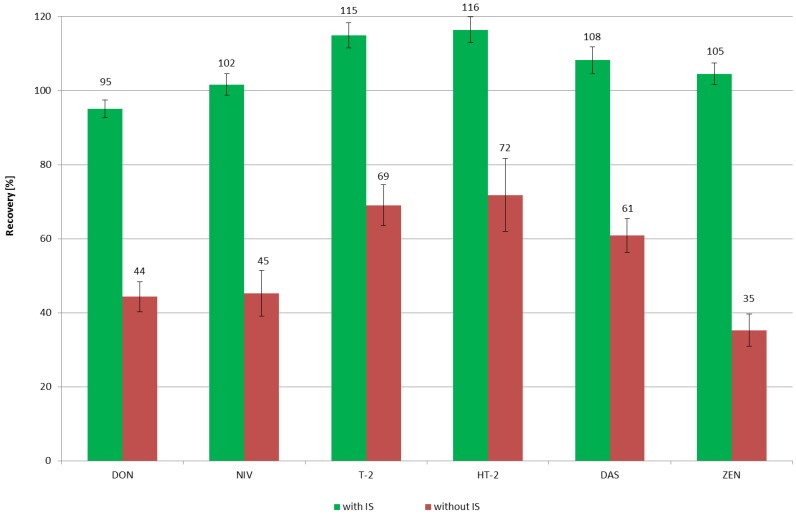
Impact of addition of internal standard (IS) on mycotoxin recovery rates.

For *Fusarium* mycotoxins, determined by the HPLC-MS/MS technique, the recovery experiments were performed both with and without the internal standards. The results showed significant differences in recovery values (Figure 1), so that only the samples with the added ISs, met the performance criteria for the analytical methods, laid down by the UE, for the official control of the levels of mycotoxins in foodstuffs [24,25]. The recovery levels, therein, were given as follows: OTA 50–120%; DON 60–110%, T-2 60–130%, HT-2 60–130%, and ZEN 60–120%. The significant differences in the recovery values, with and without ISs, confirmed the complexity of the beer matrix, resulting in significant matrix effects, especially when the applied sample clean-up procedure was not of high selectivity. The acetonitrile-based extraction caused a precipitation of polar matrix components and might also have resulted in precipitation of polar analytes, and thus decreased the recovery. Low recovery values observed in the case of ZEN, might be attributed to strong suppression caused by the co-eluting non-polar matrix [26].

### 2.2. Mycotoxins Occurrence

The results of the analyses of 69 beer samples are summarized in Table 3. OTA was detected in 93% of the samples and 78% of them contained OTA at a concentration higher than the LOQ—with the highest value at 0.347 ng/mL. In 4 beer samples (1 lager, 1 unpasteurized, and 2 porters), OTA concentrations were higher than 0.2 ng/mL; the proposed maximum acceptable level for this toxin [11].

The mean and median OTA concentrations were in the range of tens of pg/mL; these and the contamination rates are in accordance with previous European results [17,18,27,28,29]. In some traditional beers coming from Africa, however, the level of OTA might have exceeded values of 2 µg/mL [19]. DAS was not detected in any of the samples, but at least one of the *Fusarium* mycotoxins was present in 97% of samples. NIV was detected only in one sample (<LOQ), four of the samples contained ZEN (3 samples below LOQ value and 0.413 ng/mL) and the levels of T-2 and HT-2 were close to the LOQs. The mean and highest concentration of DON (detected in 96% of the samples and in 45% at the level >LOQ) were 17.1 ng/mL and 56.2 ng/mL, respectively. OTA, DON, and HT-2 were the only compounds detected in more than 75% of the samples. The results sorted by beer type are presented in Table 4.

Widespread beer contamination with DON were found, herein; confirming the results of other studies conducted in Poland and Europe [22,30,31,32]. However, differences could be observed in the prevalence of DON in beer samples. Pascari et al. [33] analyzed 64 commercially available European beers but found DON only in 4 samples (6%), whereas ZEN was present in 65% of the samples they examined; with concentrations ranging between 8.24 and 62.98 ng/mL. This is in contrast to our study where ZEN was present in only 6% of the samples, and at levels below 0.5 ng/mL. Low ZEN levels (maximum 2.0 ng/mL), similar to those herein, were also reported by Bauer et al. [31]. However, the authors there detected mycotoxin in all their samples. A selected overview of mycotoxins surveys in beer is presented in Table 5.

**Figure 2:**
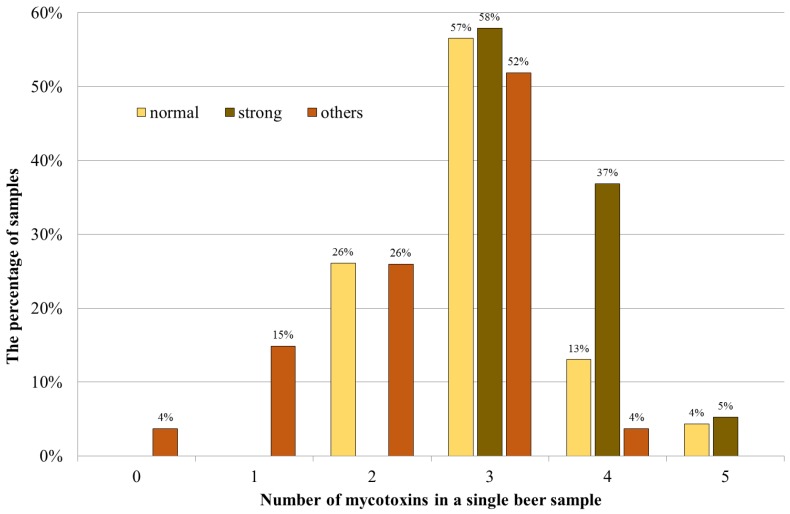
Distribution of mycotoxins in particular beer samples.

Grain and, thus, their products might be contaminated with various species of molds. Furthermore, each species can produce several mycotoxins, therefore, it is important to evaluate their co-occurrence. The association of mycotoxins might enhance the adverse health effects when compared to individual compounds [35]. More than 93% of the samples contained at least two mycotoxins, i.e., OTA and DON; and at least three compounds were present in 74% of the analyzed beers. The highest number of mycotoxins detected in one sample was 5 (3% of the samples). The distribution of mycotoxins in particular beer samples has revealed that the highest percentage, up to 60% of the samples of each group, contained 3 mycotoxins (Figure 2).

### 2.3. Dietary Exposure Assessment

Since very few of our samples were positive for mycotoxins, other than OTA, DON, and HT-2, the calculations below were only performed for those three compounds. Polish foreign trade data on beer export were used for these evaluations [36]. The Global Environmental Monitoring System (GEMS)/Food cluster diets data on mean beer consumption were as follows: Germany and Poland - 259.5mL/day; The Netherlands and Belgium—234.4; United Kingdom—180.2; USA, Canada, and Italy—174.3; Czech Republic, Hungary, Ireland, Romania, and Slovakia—225.2; and Cyprus—174.3. The mean body weight values of 70.8 kg for Europe, and 80.7 kg for Northern America were adopted for these calculations [37]. Estimated daily intake ((EDI): Table 5) was presented as a percentage of TDI (tolerable daily intake) namely: 17.14 ng/kg body-weight (b.w.) for OTA (derived from the tolerable weekly intake (TWI) = 120 ng/kg b.w.); [38], 1000 ng/kg b.w. for DON, and 60 ng/kg b.w. for HT-2 [2].

Using mean values for OTA in beer samples, the daily intake of OTA from beer approximates to 0.8–1.1% of TDI. Even a ‘worst-case’ scenario (i.e., using the maximum concentration observed, herein) only equates to 5.0–7.5% of TDI. These values are in general agreement with previous estimates from the SCOOP Task 3.2.7 [39], in which, the contribution of dietary beer to OTA intake was 0.01–0.14 ng/kg b.w. This ranks beer as a relatively small source of OTA; the contribution of other food groups was estimated as follows—cereals (44%); others (15%); wine (10%); coffee (9%); beer (7%); cocoa and derived products (5%); dried fruits (4%); meat (3%); and spices (3%). Similarly, previous studies of OTA in beer revealed that OTA intake does not exceed the TDI value [34,40].

In the case of HT-2, there were no significant differences between ‘scenarios’ (Table 6; mean—5.0–7.5% of TDI; maximum—6.5–9.7% of TDI). This was due to the fact that although HT-2 was present in 74% of the samples, its concentration was near the LOQ value of the method described herein. Rodríguez-Carrasco et al. [22] analyzed 154 beer samples for *Fusarium* mycotoxins and evaluated the average intake level of HT-2 toxin from beer to be ~7–12% of TDI, set by the Scientific Committee on Food in 2002 (0.1 μg/kg b.w.), which corresponded to ~11–20% of the TDI applied in this study.

If the mean concentration of DON from this study was used, the calculated daily exposure to this mycotoxin was in the range of 4.1–60% of TDI, whereas, based on maximum values, it reached the level of 14–21% of TDI (Table 6). This married well with previous reports, which estimated exposure to DON from beer at the level of a few percent of the TDI, for either DON [22,31] or DON with its conjugates [30,33]. Higher levels of possible exposure, close to 20% of TDI, have also been previously found [23].

## 3. Conclusions

Herein, a set of 69 beer samples was analyzed for seven mycotoxins using validated HPLC methods, relying on either fluorescence (OTA) or MS/MS (trichothecenes and ZEN) detection. Our results indicate that DON was the most frequently occurring mycotoxin, followed by the OTA and the HT-2 toxin. Moreover >93% of the beer samples examined, contained detectable levels of at least 2 analytes; and ~74% of the samples contained 3 or more mycotoxins.

Using the above data, the potential exposure of various European populations to mycotoxins from beer produced in Poland was calculated. The evidence from this analysis demonstrated that mycotoxin intake from beers made by major breweries was unlikely to exceed 20% of TDI, for any mycotoxin examined herein, and was more likely to be 5%. On one hand, we might conclude that beer did not seem to be a major source of the mycotoxins examined in this study. However, the occurrence of multiple mycotoxins in many samples and the possibility of undocumented synergies between them suggests that a cumulative mycotoxin limit might be required for beer products in the EU. In addition, the generally higher levels of mycotoxins found in craft beers suggests that these types of products should be tested more often, to protect ‘beer enthusiasts’. We also share the opinion of Varga et al. [30] and Peters et al. [34], that the maximum levels of the most frequently occurring mycotoxins found in beers should be set by the EU, in order to ensure consumer safety.

## 4. Materials and Methods

### 4.1. Sampling

Sixty-nine beer samples (from four major Polish brewing companies) were purchased randomly between January and June 2018, from retail trade in Poland, as follows—42 lager (23 normal < 6.2 abv; 19 strong > 6.2 abv), 5 porter, 9 unpasteurized, 8 flavored, and 5 non-alcoholic beers. All samples were stored at 4 °C and degassed the day before analysis.

### 4.2. Standards and Chemicals

Ochratoxin A, deoxynivalenol, nivalenol, T-2 toxin, HT-2 toxin, diacetoxyscirpenol (DAS), zearalenone (ZEN), ^13^C-DON, ^13^C-T-2, ^13^C-HT-2 toxins, and ^13^C-ZEN were purchased from Biopure (Tulln, Austria). Ammonium acetate, Celite^®^ 545, acetic acid, acetonitrile (ACN; gradient grade), and methanol (MeOH; MS grade) were supplied by Merck-Millipore (Darmstadt, Germany). The Simplicity 185 (Millipore, Bedford, MA, USA) water purification system was used for the deionized water production.

### 4.3. Sample Preparation

Before the extraction, the pH of all samples was adjusted to 7.2, by addition of 1 M NaOH. OTA was isolated using immunoaffinity columns (IAC) Ochraprep^®^ (R-Biopharm Rhône Ltd., Glasgow, UK)—30 mL of beer was passed through the column at a flow rate of 2–3 mL/min. The column was washed with 20 mL of H_2_O and dried by passing air, throughout. OTA was then eluted using 2.0 mL of MeOH:CH_3_COOH (98:2). The eluate was evaporated to dryness under nitrogen and then dissolved in 1.0 mL of H_2_O:MeOH:CH_3_COOH (50:49:1).

To prepare the samples for trichothecene and ZEN determination, 4 mL of each beer was shaken with 16 mL of ACN, 0.5 g of Celite^®^ 545, and 20 µL of ^13^C-ZEN (an internal standard for ZEN) solution for 20 min. After centrifugation (4000× *g*, 10 min), 50 µL of isotopically labelled analogues (ISs: ^13^C-DON, ^13^C-T-2, ^13^C-HT-2) were added to 5 mL of the supernatant. Samples were then evaporated to dryness under nitrogen and dissolved in 0.5 mL of MeOH:H_2_O (1:4).

### 4.4. Instrumental Analysis

OTA evaluation was performed using high-performance liquid chromatography (HPLC) with fluorescence detection (Ex: 330 nm, Em: 460 nm), using the following materials, HPLC—LaChrom ELITE HPLC system (Merck-Hitachi, Darmstadt, Germany), chromatographic column—LiChrospher 100 RP-18 (250 × 4.0 mm, 5 μm), oven temperature—25 °C, mobile phase—ACN:2%CH_3_COOH (70:30), flow rate—1 mL/min, injection volume—50 μL. 

Trichothecenes and ZEN were determined using HPLC with MS/MS detection. Analytes were separated and determined using HPLC Shimadzu Nexera (Shimadzu, Tokyo, Japan), API 4000 mass spectrometer (Sciex, Foster City, CA, USA), the Gemini–NX C18 (150 × 4.6 mm, 5 µm) (Phenomenex Inc., Torrance, CA, USA) chromatographic column, and an oven temperature of 27 °C; mobile phase of A was H_2_O + 5 mM CH_3_COONH_4_ + 1% CH_3_COOH, B: MeOH + 5 mM CH_3_COONH_4_ + 1% CH_3_COOH, flow rate was 0.7 mL/min, and the injection volume was 20 µL. HPLC-MS/MS method was performed in multiple reaction monitoring (MRM) mode in negative and positive modes, with following conditions—collision gas 6 psi, curtain gas 25 psi, ion source gas1 50 psi, ion source gas2 50 psi, ion spray voltage 5000 V (positive mode) and −4000 V (negative mode), and source temperature 500 °C. MRM analysis had two transitions per compound. Optimized analyte-dependent MS/MS parameters are given in Table 7.

The analyte identification was done, based on the retention time of the calibration solutions, as well as the spiked samples, and the signal value (area) increment, with reference to the spiking amount. For MS/MS detection, two characteristic MRM transitions for each analyte were applied as well (Table 7).

### 4.5. Statistical Analysis

To evaluate the significant differences in mycotoxin concentration among the beer types, the Kruskal-Wallis ANOVA test was applied (α = 0.05). The average values of the analytes recovered were compared, using the *t*-Student test (α = 0.05). For the calculation, the results below the LOD value were set as 0.5 LOD, whereas the results comprising between the LOD and the LOQ were set as the LOQ value for each mycotoxin. A positive sample refers to a sample with a result above the limit of detection. All statistical analyses were applied using the Statistica 10.0 software package (StatSoft, Krakow, Poland).

### 4.6. Dietary Exposure Assessment

Calculations of the estimated daily intake (EDI) of mycotoxins in different European populations were prepared, using food-consumption data derived from the GEMS/Food cluster diets 2012, prepared by WHO [41]. All contamination data (mean and median concentrations) were taken from the results of analyses conducted herein. Since beer is an alcoholic beverage, the calculation was performed for adults only; using mean body weight values adjusted for different world regions, as previously described [37]. The EDI was calculated as follows:

(1) $$\mathrm{EDI}=\frac{C\;*\;\mathrm{Cons}}{b.w.},$$

where C is the concentration of mycotoxin in contaminated beer; Cons stands for the average daily consumption of beer in the study region; and b.w. represents the body weight.

## Figures and Tables

**Table 1:** Top beer producing countries in Europe [8].

Beer Production (10^5^ L)					
Country	2012	2013	2014	2015	2016
Germany	94.6	94.4	95.3	95.6	95.0
United Kingdom	44.2	44.2	44.3	44.0	43.7
Poland	39.3	40.0	40.1	40.9	41.4
Spain	33.0	32.7	33.6	35.0	36.5
The Netherlands	24.3	23.6	23.7	24.0	24.6
France	17.6	18.3	19.9	20.3	20.7
Belgium	18.8	18.1	18.2	19.8	20.7
Czech Republic	18.7	18.7	19.1	19.5	20.5
Romania	18.0	16.1	14.8	16.0	15.8

**Table 2:** Validation data of mycotoxins from artificially contaminated beer samples.

Mycotoxin	Spiking Level [ng/mL]	Calculated Concentration [ng/mL]	Recovery [%]	Precision [%]	LOD [ng/mL]	LOQ [ng/mL]
OTA	0.05	0.0408	81.6	2.5	0.003	0.011
	0.25	0.199	79.6	6.1		
	1.0	0.858	85.8	4.2		
DON	40	38.16	95.4	2.9	3.50	11.6
	200	190.6	95.3	1.2		
	400	378.2	94.5	3.9		
NIV	40	40.84	102.1	5.2	4.30	14.3
	200	202.2	101.1	1.7		
	400	407.2	101.8	1.3		
T-2	8	9.36	117.0	3.9	0.31	1.03
	40	44.64	111.6	3.0		
	80	92.8	116.0	1.3		
HT-2	8	9.10	113.8	5.2	0.36	1.21
	40	47.64	119.1	3.0		
	80	93.04	116.3	1.2		
DAS	8	8.72	109.0	4.0	0.28	0.92
	40	43.92	109.8	2.6		
	80	84.64	105.8	4.1		
ZEN	4	4.14	103.5	3.4	0.07	0.23
	20	20.74	103.7	2.5		
	40	42.56	106.4	3.0		

**Table 3:** Concentration of ochratoxin A (OTA) and *Fusarium* mycotoxins in the analyzed beer samples.

Toxin	Positive/Samples	Mean [ng/mL]	Median [ng/mL]	Positive Samples [ng/mL]		
				Min	Max	Mean; SD
OTA	64/69	0.053	0.032	<LOQ	0.347	0.057; 0.065
DON	66/69	16.3	<LOQ	<LOQ	56.2	17.1; 9.0
NIV	1/69	<LOD	<LOD	-	<LOQ	-
T-2	11/69	<LOD	<LOD	<LOQ	<LOQ	<LOQ
HT-2	51/69	<LOQ	<LOQ	<LOQ	1.57	1.23; 0.08
DAS	0/69	-	-	-	<LOD	-
ZEN	4/69	<LOD	<LOD	<LOQ	0.413	0.304; 0.073

**Table 4:** Occurrence of OTA, DON, and HT-2 in different beer type samples.

	Beer Type	Positive/Samples	Mean [ng/mL]	Median [ng/mL]	Positive Samples [ng/mL]		
					Min	Max	Mean
OTA	Normal lager	23/23	0.051	0.034	<LOQ	0.347	0.051
	Strong lager	19/19	0.062	0.056	0.012	0.114	0.062
	Porter	5/5	0.111	0.068	<LOQ	0.262	0.112
	Unpasteurized	8/9	0.059	0.023	<LOQ	0.269	0.066
	Flavored	5/8	0.017	0.014	<LOQ	0.046	0.027
	Non-alcoholic	4/5	0.013	<LOQ	<LOQ	0.030	0.016
DON	Normal lager	22/23	17.9	<LOQ	<LOQ	56.2	18.7
	Strong lager	19/19	19.2	18.8	<LOQ	34.7	19.2
	Porter	4/5	16.5	<LOQ	<LOQ	33.8	20.7
	Unpasteurized	9/9	13.4	<LOQ	<LOQ	16.3	13.4
	Flavored	8/8	12.3	<LOQ	<LOQ	16.7	12.3
	Non-alcoholic	4/5	<LOQ	<LOQ	<LOQ	12.7	11.9
HT-2	Normal lager	17/23	<LOQ	<LOQ	<LOQ	1.57	1.23
	Strong lager	19/19	1.23	<LOQ	<LOQ	1.52	1.23
	Porter	4/5	<LOQ	<LOQ	<LOQ	1.52	1.29
	Unpasteurized	6/9	<LOQ	<LOQ	<LOQ	<LOQ	<LOQ
	Flavored	3/8	<LOQ	<LOD	<LOQ	<LOQ	<LOQ
	Non-alcoholic	2/5	<LOQ	<LOD	<LOQ	<LOQ	<LOQ

**Table 5:** Overview of mycotoxin occurrence in beer samples.

Mycotoxin	Number of Analyzed Beer Samples	Number of Positive Samples	Mycotoxin Concentration Range (ng/mL)	Reference
OTA	69	69	0.008–0.498	[18]
	19	10	1.5–2340	[19]
	318	233	0.01–0.293	[27]
	88	73	0.007–0.204	[29]
	1000	6	<0.3–0.6	[34]
DON	154	60	24.5–47.7	[22]
	374	204	3.2–89.3	[30]
	44	33	2.2–20.0	[31]
	100	83	1.0–73.6	[32]
	64	4	20.97–46.74	[33]
	1000	64	<10–412	[34]
NIV	100	56	0.5–7.6	[32]
	1000	4	8–21	[34]
T-2	1000	3	<0.5–2.3	[34]
HT-2	154	14	24.2–38.2	[22]
	64	1	23.72	[33]
	1000	1	3.4	[34]
ZEN	35	7	2.6–426	[19]
	64	12	8.24–62.96	[33]
	44	44	0.35–2.0	[31]
	1000	6	<0.3–0.3	[34]

**Table 6:** Estimated daily intake (EDI) of OTA, DON, and HT-2 derived from the determined mycotoxin level, beer consumption in various European countries [41], mean body weight [37], and tolerable daily intake (TDIs) [2,38].

Cluster	Country	Toxin Level	EDI [ng/kg b.w.]			% TDI		
			OTA	DON	HT-2	OTA	DON	HT-2
G07	UK	mean	0.14	42.0	3.11	0.80	4.20	5.19
		maximum	0.89	145	4.00	5.21	14.5	6.74
G08	Germany Poland	mean	0.20	60.4	4.48	1.14	6.04	7.47
		maximum	1.28	208	5.80	7.50	20.8	9.69
G10	Canada Italy USA	mean	0.13	40.6	3.01	0.77	4.06	5.02
		maximum	0.86	140	3.90	5.04	14.0	6.52
G15	Czech Republic Hungary Ireland Romania Slovakia	mean	0.17	52.4	3.89	0.99	5.24	6.49
		maximum	1.12	181	5.10	6.51	18.1	8.42

**Table 7:** Optimized mass spectrometry conditions.

Compound	Precursor Ion (m/z)	Declustering Potential (V)	Product Ions (m/z) ^a^	Collision Energy (V)	Cell Exit Potential (V)
Nivalenol	371.1 [M + CH_3_COO]^−^	−40	**281.0**/59.0	−22/−40	−14/−5
Deoxyniwalenol	355.1 [M + CH_3_COO]^−^	−35	**264.8**/58.9	−20/−38	−17/−1
13C-Deoxyniwalenol	370.2 [M + CH_3_COO]^−^	−50	310.0	−14	−7
Diacetoxyscirpenol	384.1 [M + NH_4_]^+^	51	**307.0**/247.0	17/19	20/16
T-2 toxin	484.1 [M + NH_4_]^+^	61	**215.0**/185.0	29/25	12/14
13C-T-2 toxin	508.3 [M + NH_4_]^+^	61	322.1	19	8
HT-2 toxin	442.2 [M + NH_4_]^+^	51	**215.0**/263.0	19/17	14/18
13C-HT-2 toxin	464.1 [M + NH_4_]^+^	51	278.1	17	18
Zearalenone	317.1 [M − H]^−^	−85	**130.8**/174.9	−40/−32	−7/−9
13C-Zearalenone	355.1 [M − H]^−^	−100	139.9	−42	−7

^a^ quantifier ions are given in bold.

## Abbreviations

Abv	alcohol by volume
ACN	acetonitrile
b.w.	body-weight
DAS	diacetoxyscirpenol
DON	deoxynivalenol
EDI	estimated daily intake
HPLC	high performance liquid chromatography
ISs	internal standards
LOD	limit of detection
LOQ	limit of quantitation
NIV	nivalenol
OTA	ochratoxin A
TDI	tolerable daily intake
ZEN	zearalenone

## References

[1] Hornsey I. (2003). A History of Beer and Brewing.

[2] European Commission (2006). Commission regulation (EC) No 1881/2006 of 19 December 2006 setting maximum levels for certain contaminants in foodstuffs. Off. J. Eur. Union.

[3] European Commission (2007). Commission regulation (EC) No 1126/2007 of 28 September 2007 amending Regulation (EC) no 1881/2006 setting maximum levels for certain contaminants in foodstuffs as regards *Fusarium* toxins in maize and maize products. Off. J. Eur. Union.

[4] European Commission (2010). Commission regulation (EU) No 105/2010 of 5 February 2010 amending regulation (EC) no 1881/2006 setting maximum levels for certain contaminants in foodstuffs as regards ochratoxin A. Off. J. Eur. Union.

[5] European Commission (2010). Commission regulation (EU) No 165/2010 of 26 February 2010 amending Regulation (EC) no 1881/2006 setting maximum levels for certain contaminants in foodstuffs as regards aflatoxins. Off. J. Eur. Union.

[6] European Commission (2012). Commission regulation (EU) No 594/2012 of 5 July 2012 amending Regulation (EC) 1881/2006 as regards the maximum levels of the contaminants ochratoxin A, non dioxin-like PCBs and melamine in foodstuffs. Off. J. Eur. Union.

[7] European Commission (2013). Commission recommendation of 27 March 2013 on the presence of T-2 and HT-2 toxin in cereals and cereal products. Off. J. Eur. Union.

[8] (2017). Beer Production 2010–2016. Beer Statistics 2017.

[9] Milićević D.R., Škrinjar M., Baltić T. (2010). Real and perceived risks for mycotoxin contamination in foods and feeds: Challenges for food safety control. Toxins.

[10] Bayman P., Baker J.L. (2006). Ochratoxins: A global perspective. Mycopathologia.

[11] Anli E., Mert Alkis I. (2010). Ochratoxin A and brewing technology: A review. J. Inst. Brew..

[12] Lancova K., Hajslova J., Poustka J., Krplova A., Zachariasova M., Dostalek P., Sachambula L. (2008). Transfer of *Fusarium* mycotoxins and ‘masked’ deoxynivalenol (deoxynivalenol-3-glucoside) from field barley through malt to beer. Food Addit. Contam..

[13] Kostelanska M., Zachariasova M., Lacina O., Fenclova M., Kollos A.L., Hajslova J. (2011). The study of deoxynivalenol and its masked metabolites fate during the brewing process realised by UPLC-TOFMS method. Food Chem..

[14] Wolf-Hall C.E. (2007). Mold and mycotoxin problems encountered during malting and brewing. Int. J. Food Microbiol..

[15] Habschied K., Šarkanj B., Klapec T., Krstanović V. (2011). Distribution of zearalenone in malted barley fractions dependent on *Fusarium* graminearum growing conditions. Food Chem..

[16] Bĕláková S., Benešová K., Čáslavský J., Svoboda Z. (2014). The occurrence of the selected *Fusarium* mycotoxins in Czech malting barley. Food Control..

[17] Jørgensen K. (1998). Survey of pork, poultry, coffee, beer and pulses for ochratoxin A. Food Addit. Contam..

[18] Medina A., Valle-Algarra F.M., Gimeno-Adelantado J.V., Mateo R., Mateo F., Jiménez M. (2006). New method for determination of ochratoxin A in beer using zinc acetate and solid-phase extraction silica cartridges. J. Chromatogr. A.

[19] Odhav B., Naicker V. (2002). Mycotoxins in South African traditionally brewed beers. Food Addit. Contam..

[20] Zöllner P., Berner D., Jodlbauer J., Lindner W. (2000). Determination of zearalenone and its metabolites alpha- and beta-zearalenol in beer samples by high-performance liquid chromatography-tandem mass spectrometry. J. Chromatogr. B Biomed. Sci. Appl..

[21] Schothorst R.C., Jekel A.A. (2003). Determination of trichothecenes in beer by capillary gas chromatography with flame ionisation detection. Food Chem..

[22] Rodríguez-Carrasco Y., Fattore M., Albrizio S., Berrada H., Mañes J. (2015). Occurrence of *Fusarium* mycotoxins and their dietary intake through beer consumption by the European population. Food Chem..

[23] Piacentini K.C., Savi G.D., Olivo G., Scussel V.M. (2015). Quality and occurrence of deoxynivalenol and fumonisins in craft beer. Food Control.

[24] European Commission (2006). Commission regulation (EC) No 401/2006 of 23 February 2006 laying down the methods of sampling and analysis for the official control of the levels of mycotoxins in foodstuffs. Off. J. Eur. Union.

[25] European Commission (2014). Commission regulation (EU) No 519/2014 of 16 May 2014 amending Regulation (EC) No 401/2006 as regards methods of sampling of large lots, spices and food supplements, performance criteria for T-2, HT-2 toxin and citrinin and screening methods of analysis. Off. J. Eur. Union.

[26] Habler K., Gotthardt M., Schüler J., Rychlik M. (2017). Multi-mycotoxin stable isotope dilution LC-MS/MS method for *Fusarium* toxins in beer. Food Chem..

[27] Bresch H., Urbanek M., Hell K. (2000). Ochratoxin A in coffee, tea and beer. Arch. Fur Lebensm..

[28] Tangni E.K., Ponchaut S., Maudoux M., Rozenberg R., Larondelle Y. (2002). Ochratoxin A in domestic and imported beers in Belgium: Occurrence and exposure assessment. Food Addit. Contam..

[29] Medina A., Jiménez M., Gimeno-Adelantado J.V., Valle-Algarra F.M., Mateo R. (2005). Determination of ochratoxin A in beer marketed in Spain by liquid chromatography with fluorescence detection using lead hydroxyacetate as a clean-up agent. J. Chromatogr. A.

[30] Varga E., Malachowa A., Schwartz H., Krska R., Berthiller F. (2013). Survey of deoxynivalenol and its conjugates deoxynivalenol-3-glucoside and 3-acetyl-deoxynivalenol on 374 beer samples. Food Addit. Contam..

[31] Bauer J.I., Gross M., Gottschalk C., Usleber E. (2016). Investigations on the occurrence of mycotoxins in beer. Food Control.

[32] Bryła M., Ksieniewicz-Woźniak E., Waśkiewicz A., Szymczyk K., Jędrzejczak R. (2018). Co-occurrence of nivalenol, deoxynivalenol and deoxynivalenol-3-glucoside in beer samples. Food Control..

[33] Pascari X., Ortiz-Solá J., Marín S., Ramos A.J., Sanchis V. (2018). Survey of mycotoxins in beer and exposure assessment through the consumption of commercially available beer in Lleida, Spain. LWT Food Sci. Technol..

[34] Peters J., van Dam R., van Doorn R., Katerere D., Berthiller F., Haasnoot W., Nielen M.W.F. (2017). Mycotoxin profiling of 1000 beer samples with a special focus on craft beer. PLoS ONE.

[35] Grenier B., Oswald I.P. (2011). Mycotoxin co-contamination of food and feed: Meta-analysis of publications describing toxicological interactions. World Mycotoxin J..

[36] (2017). The Yearbook of Foreign Trade Statistics of the Polish Central Statistical Office, Warsaw. https://stat.gov.pl/obszary-tematyczne/roczniki-statystyczne/roczniki-statystyczne/rocznik-statystyczny-handlu-zagranicznego-2017,9,11.html.

[37] Walpole S.C., Prieto-Merino D., Edwards P., Cleland J., Stevens G., Roberts I. (2012). The weight of nations: An estimation of adult human biomass. BMC Public Health.

[38] EFSA (European Food Safety Authority) (2010). Statement on recent scientific information on the toxicity of Ochratoxin A. EFSA J..

[39] European Commission Task 3.2.7 (2002). Assessment of dietary intake of Ochratoxin A by the population of EU Member States. https://ec.europa.eu/food/safety/chemical_safety/contaminants/catalogue/ochratoxin_en.

[40] Coronel M.B., Marín S., Cano-Sancho G., Ramos A.J., Sanchis V. (2012). Exposure assessment to ochratoxin A in Catalonia (Spain) based on the consumption of cereals, nuts, coffee, wine, and beer. Food Addit. Contam. Part A.

[41] GEMS/Food Consumption Cluster Diets (2012). Global Environment Monitoring System Food Contamination Monitoring and Assessment Programme. https://extranet.who.int/sree/Reports?op=vs&path=/WHO_HQ_Reports/G7/PROD/EXT/GEMS_cluster_diets_2012&userid=G7_ro&password=inetsoft123.

